# Hypomorphic conditional deletion of E11/Podoplanin reveals a role in osteocyte dendrite elongation

**DOI:** 10.1002/jcp.25999

**Published:** 2017-06-14

**Authors:** Katherine A. Staines, Behzad Javaheri, Peter Hohenstein, Robert Fleming, Ekele Ikpegbu, Erin Unger, Mark Hopkinson, David J. Buttle, Andrew A. Pitsillides, Colin Farquharson

**Affiliations:** ^1^ Roslin Institute and R(D)SVS The University of Edinburgh, Easter Bush Midlothian UK; ^2^ School of Applied Sciences Edinburgh Napier University, Sighthill Campus Edinburgh UK; ^3^ Comparative Biomedical Sciences Royal Veterinary College London UK; ^4^ Michael Okpara University of Agriculture Nigeria; ^5^ Department of Infection, Immunity and Cardiovascular Disease University of Sheffield Sheffield UK

**Keywords:** dendrite, E11, osteoblasts, osteocalcin, osteocytes

## Abstract

The transmembrane glycoprotein E11/Podoplanin (Pdpn) has been implicated in the initial stages of osteocyte differentiation. However, its precise function and regulatory mechanisms are still unknown. Due to the known embryonic lethality induced by global *Pdpn* deletion, we have herein explored the effect of bone‐specific *Pdpn* knockdown on osteocyte form and function in the post‐natal mouse. Extensive skeletal phenotyping of male and female 6‐week‐old *Oc*‐cre;*Pdpn* ^flox/flox^ (cKO) mice and their *Pdpn* ^flox/flox^ controls (fl/fl) has revealed that *Pdpn* deletion significantly compromises tibial cortical bone microarchitecture in both sexes, albeit to different extents (*p* < 0.05). Consistent with this, we observed an increase in stiffness in female cKO mice in comparison to fl/fl mice (*p* < 0.01). Moreover, analysis of the osteocyte phenotype by phalloidin staining revealed a significant decrease in the dendrite volume (*p* < 0.001) and length (*p* < 0.001) in cKO mice in which deletion of *Pdpn* also modifies the bone anabolic loading response (*p* < 0.05) in comparison to age‐matched fl/fl mice. Together, these data confirm a regulatory role for *Pdpn* in osteocyte dendrite formation and as such, in the control of osteocyte function. As the osteocyte dendritic network is known to play vital roles in regulating bone modeling/remodeling, this highlights an essential role for *Pdpn* in bone homeostasis.

## 1 INTRODUCTION

Osteocytes are the most numerous of all the cells within bone and are critical regulators of bone structure and function. Most original paradigms have proposed that osteocytes are passive in their formation (Nefussi, Sautier, Nicolas, & Forest, 1991; Palumbo, Ferretti, & Marotti, 2004; Skerry, Bitensky, Chayen, & Lanyon, 1989). Recent evidence, however, now indicates that initial “embedding” involves their active contribution with vital transcriptional and dramatic morphological transformations (Holmbeck et al., 2005). Osteocytes have a highly specialized morphology with numerous dendritic processes extending from their cell body, connecting them with other osteocytes and with cells lining the bone surfaces, creating a large syncytium to enable cell–cell communications. Osteocytes play an integral role in maintaining bone homeostasis by regulating bone modeling and remodeling through their production of the Wnt inhibitor sclerostin, and through communicating with bone‐resorbing osteoclasts by RANKL production (Balemans et al., 2001; Dallas, Prideaux, & Bonewald, 2013; Nakashima & Takayanagi, 2011; Xiong et al., 2011).

Although it is well recognized that osteocytes are derived from osteoblasts, the mechanisms which govern this transition (osteocytogenesis) are yet to be fully elucidated. Several different genes have been implicated in influencing osteocytogenesis, one of which encodes for E11/Podoplanin (Pdpn) (Zhang et al., 2006). Pdpn is a mucin‐like, transmembrane glycoprotein, which undergoes O‐glycosylation leading to the production of different glycoforms. Pdpn is up‐regulated by hypoxia in the lung (Cao, Ramirez, & Williams, 2003), IL‐3 and PROX‐1 in the lymphatic system (Groger et al., 2004; Hong et al., 2002), and by TGF‐β in fibrosarcoma cells (Suzuki et al., 2005).

Few studies have investigated the precise function of Pdpn in osteocytes. We have previously shown that Pdpn is expressed by early embedding osteocytes, thus identifying it as a factor which likely contributes to the vital, early stages of osteocyte differentiation (Staines, Prideaux, et al., 2016). It is known that *Pdpn* expression in osteocytes is up‐regulated in response to mechanical strain in vivo (Zhang et al., 2006) and that increased Pdpn expression, through overexpression in ROS 17/2.6 cells and through stabilization by proteasome inhibitors in MLO‐A5 cells, leads to the formation of long dendritic processes (Sprague, Wetterwald, Heinzman, & Atkinson, 1996; Staines, Prideaux, et al., 2016). The formation of these cytoplasmic processes is abrogated in cells pre‐treated with siRNA targeted against *Pdpn* (Zhang et al., 2006). Although these data support an important role for Pdpn in dendritic process formation, a key feature of the differentiating osteocyte, the underpinning mechanisms remain to be fully defined.

While in vitro studies are informative, to fully disclose the biological function of Pdpn during in vivo osteocytogenesis and bone modeling/remodeling, it is essential to study an in vivo model of *Pdpn* deletion to enable a thorough examination of its functional role. Global deletion of *Pdpn* is perinatally lethal due to the essential role of Pdpn in lung and epithelial cell function (Zhang et al., 2006). We have therefore generated a *Pdpn* conditional knockout mouse to examine how deletion of *Pdpn* influences osteocytogenesis and the skeletal phenotype of these mice. We have used the well characterized osteocalcin (OC)‐cre promotor mouse (Zhang et al., 2002) as the expression of osteocalcin by late osteoblasts ensures that we eliminate Pdpn expression from late osteoblasts as they transition to the osteocyte phenotype. Using an osteocyte‐specific cre mouse would have invalidated our experimental approach as it would have precluded our ability to study the role of Pdpn in osteocyte formation; osteoblasts would already have undergone differentiation into osteocytes. It is also pertinent to add that an osteocyte‐specific cre‐promotor mouse does not currently exist. Here, we show that a significant reduction in the expression of *Pdpn* in mice affects tibial microarchitecture, compromises osteocyte dendrite elongation, and thereby implicating Pdpn as a regulator of both osteocyte form and function.

## 2 RESULTS

### 2.1 Oc‐cre mediated bone‐specific deletion of Pdpn

**Figure 1:**
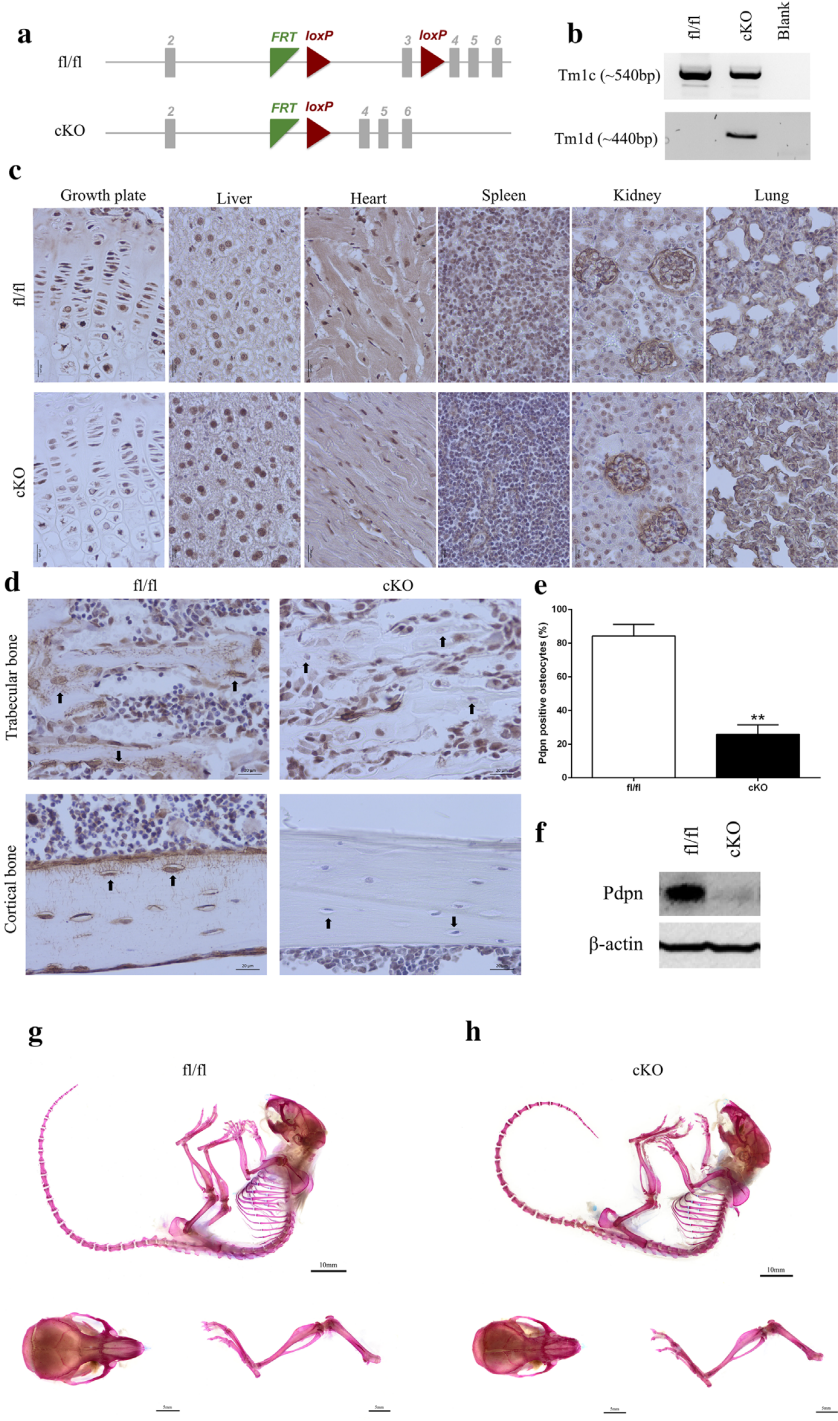
(a) Schematic of the *Pdpn* floxed allele before and after deletion of the loxP cassette containing exon 3 via osteocalcin cre (*Oc*‐cre) mediated recombination. (b) PCR analysis of genomic DNA from the long bones of fl/fl, and cKO mice with primers for the Tm1c allele (before cre recombination), and the Tm1d allele (∼440 bp, after cre recombination). (c) Immunohistochemical labeling of Pdpn in the lung, kidney, spleen, heart, liver, muscle, and growth plate, of 6‐week‐old mice. Images are representative of *n* = 4/sex/genotype. Scale bar = 20 μm. (d) Immunohistochemical labeling of Pdpn in the trabecular bone and cortical bone of 6‐week‐old mice. Arrows are pointing at embedded osteocytes within the trabecular and cortical bone and their dendritic processes projecting from the cell bodies. Images are representative of *n* = 4/sex/genotype. Scale bar = 20μm. (e) Quantification of osteocytes positive for Pdpn immunolabeling relative to negatively labeled osteocytes (*n* = 3/genotype), *p* < 0.001***. (f) Western blotting for Pdpn (∼37 kDa) in cortical bone protein lysates from 6‐week‐old mice. β‐actin was used as a loading control. Whole mount Alcian Blue and Alizarin Red stained skeletal preparations of 6‐week‐old male (g) fl/fl, and (h) cKO mice (scale bar = 10 mm) including hindlimb and calvaria preparations (scale bar = 5 mm)

The genotypes from the breeding strategy were born at the expected Mendelian frequency, and all cKO mice exhibited survival indistinguishable from that of fl/fl control mice. To confirm that the *Pdpn* floxed allele was selectively deleted in bone in cKO mice, we performed PCR analysis using primers designed to specifically detect *Pdpn* alleles before (Tm1c) and after (Tm1d) cre‐recombination. As expected, the Tm1d allele was only present in the cKO mice revealing selective deletion of *Pdpn* in bone (Figure 1b). The presence of reduced levels of the retained Tm1c allele in the bones from cKO mice indicates that complete *Pdpn* deletion was, however, not achieved (Figure 1b). This bone‐selective hypomorphic expression of *Pdpn* in cKO mice was confirmed by immunohistochemical and immunoblotting data. In both genotypes, immunolabeling for Pdpn showed extensive expression throughout all soft tissues including the lung, kidney, and liver (Figure 1c). Pdpn immunolabeling was also observed in the growth plate chondrocytes of all mice (Figure 1c). Osteoblasts in both the fl/fl and the cKO mice also stained positively for Pdpn. In the trabecular and cortical bone of the fl/fl mice, osteocytes and their dendritic processes exhibited positive immunolabeling for Pdpn (Figure 1d). However, in the cKO mice, approximately 70% of osteocytes did not label positively for Pdpn, thereby indicating a significant reduction in bone Pdpn in these cKO mice (*p* < 0.001; Figure 1d,e). Similarly, Western blotting revealed little Pdpn expression in the bones from cKO compared to fl/fl mice (Figure 1f). Together, these data confirm the hypomorphic loss of *Pdpn* expression selectively from bone in our cKO mice.

### 2.2 Bone‐selective ablation of Pdpn has no effect on the gross skeletal phenotype

Analysis of total body weight (g) of male or female 6‐week‐old mice showed no significant differences between genotypes (male fl/fl: 20.4 ± 0.5; male cKO: 19.1 ± 0.4; female fl/fl: 16.3 ± 0.6; female cKO: 16.2 ± 0.5; n>4/genotype/sex). Similarly, no differences were observed in the tibia lengths (mm) in cKO and fl/fl mice (male fl/fl: 16.2 ± 0.1; male cKO: 16.1 ± 0.2; female fl/fl: 15.6 ± 0.2; female cKO: 15.5 ± 0.1; *n* > 4/genotype/sex). Whole mount skeletal staining of mice also revealed no obvious gross differences in Alcian blue or Alizarin Red staining between cKO and fl/fl mice (Figure 1g,h). Gait parameters of freely moving mice using the CatWalk gait analysis system were also unchanged (Supplementary Figure S1). Together these data suggest that the bone‐selective deletion of *Pdpn* has no effect on the gross skeletal phenotype of mice.

### 2.3 Pdpn deletion significantly alters tibial cortical bone microarchitecture

**Figure 2:**
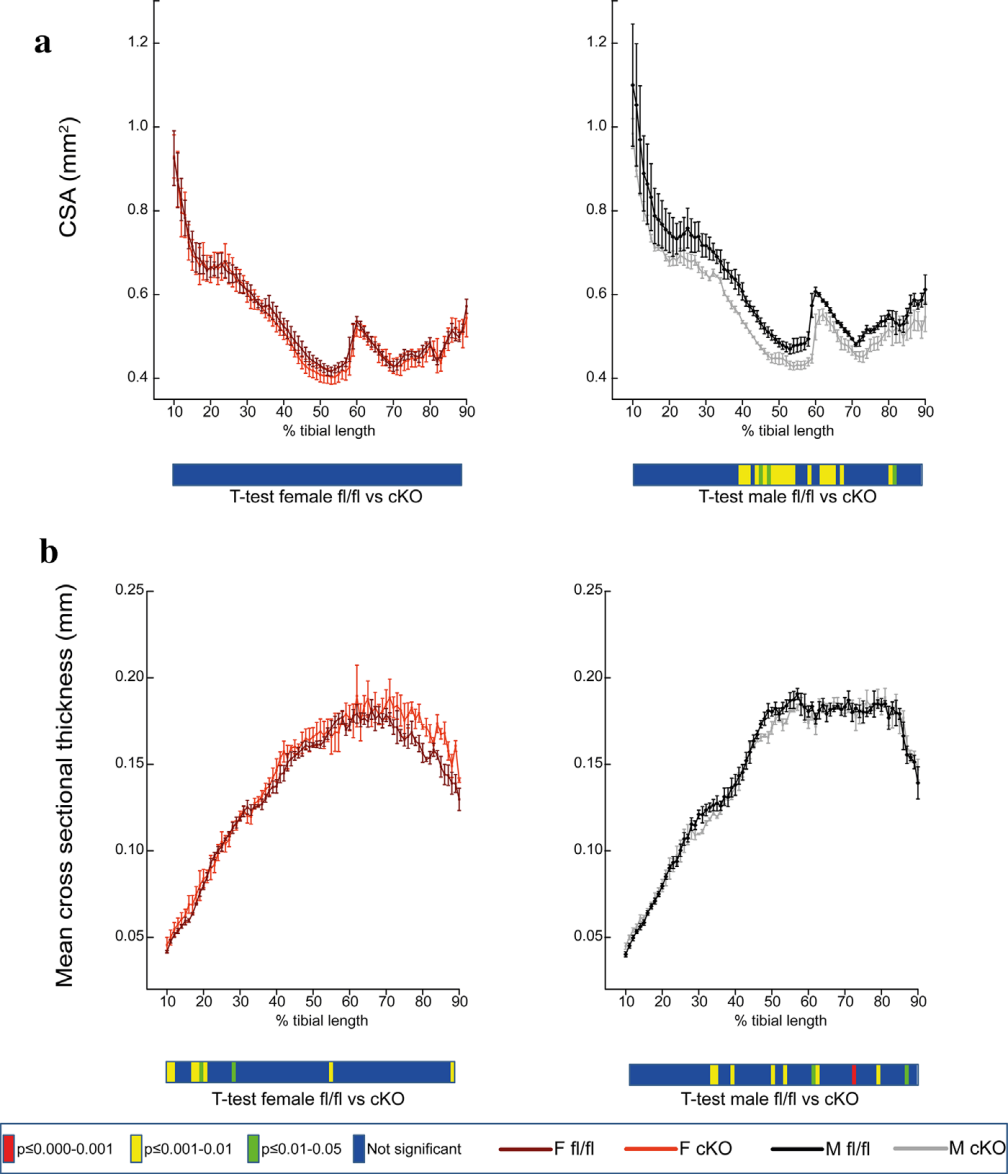
Whole bone analyses of cortical bone between 10% and 90% of total tibial length, excluding proximal and distal metaphyseal bone, of female fl/fl (brown), female cKO (orange), male fl/fl (black), and male cKO (gray) tibia at 6 weeks of age showing (a) cross‐sectional area (CSA; mm^2^) and (b) mean cross‐sectional thickness (mm). Graphs represent mean ± SEM, *n* = 4/group. *p* < 0.05 was considered to be significant and *p* ≤ 0.01–0.05 was noted as green, *p* ≤ 0.001–0.01 as yellow, and *p* ≤ 0.000–0.001 as red. Not significant is noted as blue

Assessment of cortical bone mass by μCT (CSA/mean cortical thickness) revealed that *Pdpn* deletion produces statistically significant alterations in tibial cortical mass and shape, to differing extents, in both male and female cKO compared to fl/fl control mice. Specifically, CSA is unaffected in female cKO mice but is significantly lower in male cKO compared with male fl/fl mice at ∼40–65% of tibial length (*p* < 0.05; Figure 2a). In addition, mean cross‐sectional thickness was significantly lower at several regions in male cKO compared to male fl/fl mice (*p* < 0.05); however, *Pdpn* deletion resulted in an increase in mean cross‐sectional thickness at several regions along the tibial length in female cKO compared with fl/fl control mice (*p* < 0.05; Figure 2b). Together these data indicate that the conditional deletion of *Pdpn* produces a deficit in the cortical tibial microarchitecture in both male and female mice, with a greater effect observed in the male mice. Despite this, no significant differences were observed in the trabecular bone volume/tissue volume (BV/TV), trabecular number (Tb.N), trabecular thickness (Tb.th), trabecular separation (Tb.Sp), or trabecular pattern factor (Tb.Pf) in male or female cKO mice in comparison to age‐matched fl/fl control mice (Table 1).

**Table 1:** Trabecular parameters from microCT analysis of 6‐week‐old male and female fl/fl and cKO mice (*n* = 4/genotype/sex)

Sex		BV/TV (%)	Tb.N (μm^−1^)	Tb.Th (μm)	Tb.Sp (μm)	Tb.Pf (μm^−1^)
M	fl/fl	0.40 ± 0.05	1.14 ± 0.06	28.1 ± 3.53	1.19 ± 0.06	9.84 ± 3.71
	cKO	0.38 ± 0.03	1.12 ± 0.07	30.3 ± 1.57	1.11 ± 0.09	10.6 ± 3.12
F	fl/fl	0.23 ± 0.03^*^	1.19 ± 0.02	32.7 ± 1.65	0.96 ± 0.08	5.96 ± 3.05
	cKO	0.23 ± 0.03	1.07 ± 0.03	33.2 ± 3.36	1.12 ± 0.08	9.54 ± 2.68

Data are represented as mean ± SEM.

^*^ *p* < 0.05 in comparison to male equivalent genotype.

### 2.4 Hypomorphic deletion of Pdpn results in gender‐dependent effects on tibial structural parameters

**Figure 3:**
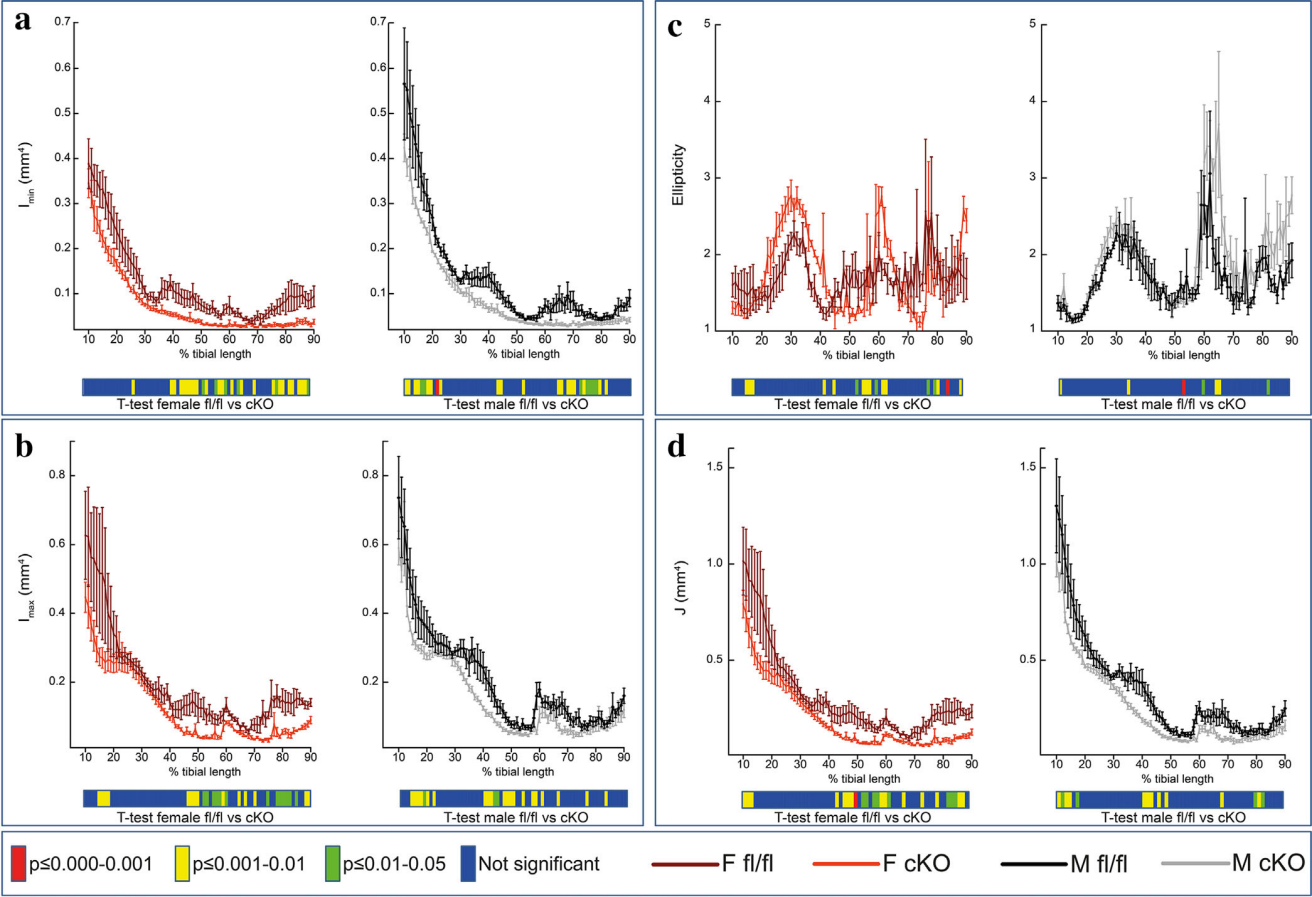
Whole bone analyses of cortical bone between 10% and 90% of total tibial length, excluding proximal and distal metaphyseal bone, of female fl/fl (brown), female cKO (orange), male fl/fl (black), and male cKO (gray) tibia at 6 weeks of age showing (a) I_min_ (mm^4^), (b) I_max_ (mm^4^), (c) ellipticity, and (d) resistance to torsion (J; mm^4^) Graphs represent mean ± SEM, *n* = 4/group. *p* < 0.05 was considered to be significant and *p* ≤ 0.01–0.05 was noted as green, *p* ≤ 0.001–0.01 as yellow, and *p* ≤ 0.000–0.001 as red. Not significant is noted as blue

To provide an estimate of tibial resistance to bending forces, we calculated second moment of area around minor (I_min_) and major axes (I_max_) (Figure 3a,b). This showed a reduction in I_min_ along the tibia length in female cKO compared to female fl/fl which was most pronounced in midshaft and distal tibia (*p* < 0.05, Figure 3a). Statically significant reduction in I_min_ was also apparent in the tibia of male cKO compared with their male fl/fl controls, less so at the midshaft but more proximally (*p* < 0.05, Figure 3a). Overall, the trend in cKO mice was for smooth lowering of I_min_ proximodistally, which contrasts markedly from fluctuations in I_min_ along the tibia length of age‐matched fl/fl control mice. For I_max_, the trend was similar for female cKO and their fl/fl controls (*p* < 0.05, Figure 3b); however, the effect in male cKO seemed to be more proximal and midshaft (*p* < 0.05, Figure 3b). Tibial ellipticity and resistance to torsion (J) was mostly affected in female cKO compared with female fl/fl (*p* < 0.05, Figure 3c,d) with only small regions affected in male cKO compared with their male controls (*p* < 0.05, Figure 3c,d). This suggests that tibiae in female cKO are weaker than in fl/fl control mice. Indeed our 3‐point bending data show a 52% increase in stiffness in the female cKO in comparison to fl/fl mice (*p* < 0.01; Table 2). No other significant differences were observed in bone strength, work to rupture, or maximum load between genotypes in either gender (Table 2).

**Table 2:** 3‐point bending analysis of 6‐week‐old male and female fl/fl and cKO mice (*n* = 4/genotype/sex)

Sex		Maximum load (N)	Deflection at maximum load (mm)	Work to maximum load (J)	Stiffness (N/m)	Load at rupture (N)	Deflection at rupture (mm)	Work to rupture (J)
M	fl/fl	5.81 ± 0.65	0.60 ± 0.09	0.002 ± 0.00	17.7 ± 0.66	4.06 ± 0.45	0.80 ± 0.14	0.003 ± 0.00
	cKO	5.95 ± 0.26	0.78 ± 0.14	0.002 ± 0.00	16.4 ± 0.97	4.22 ± 0.19	1.08 ± 0.16	0.004 ± 0.00
F	fl/fl	4.58 ± 0.34	0.66 ± 0.04	0.002 ± 0.00	12.6 ± 1.42	3.21 ± 0.24^*^	0.90 ± 0.02	0.002 ± 0.00
	cKO	5.30 ± 0.51	0.68 ± 0.06	0.002 ± 0.00	19.1 ± 1.44^**^	3.88 ± 0.36	0.96 ± 0.11	0.003 ± 0.00

Data are represented as mean ± SEM.

^*^ *p* < 0.05 in comparison to male equivalent genotype.

^**^ *p *< 0.01 in comparison to female fl/fl. Data are represented as mean ± S.E.M.

### 2.5 Bone selective Pdpn hypomorphism does not affect osteocyte differentiation but causes disruption of the osteocyte dendritic network

**Figure 4:**
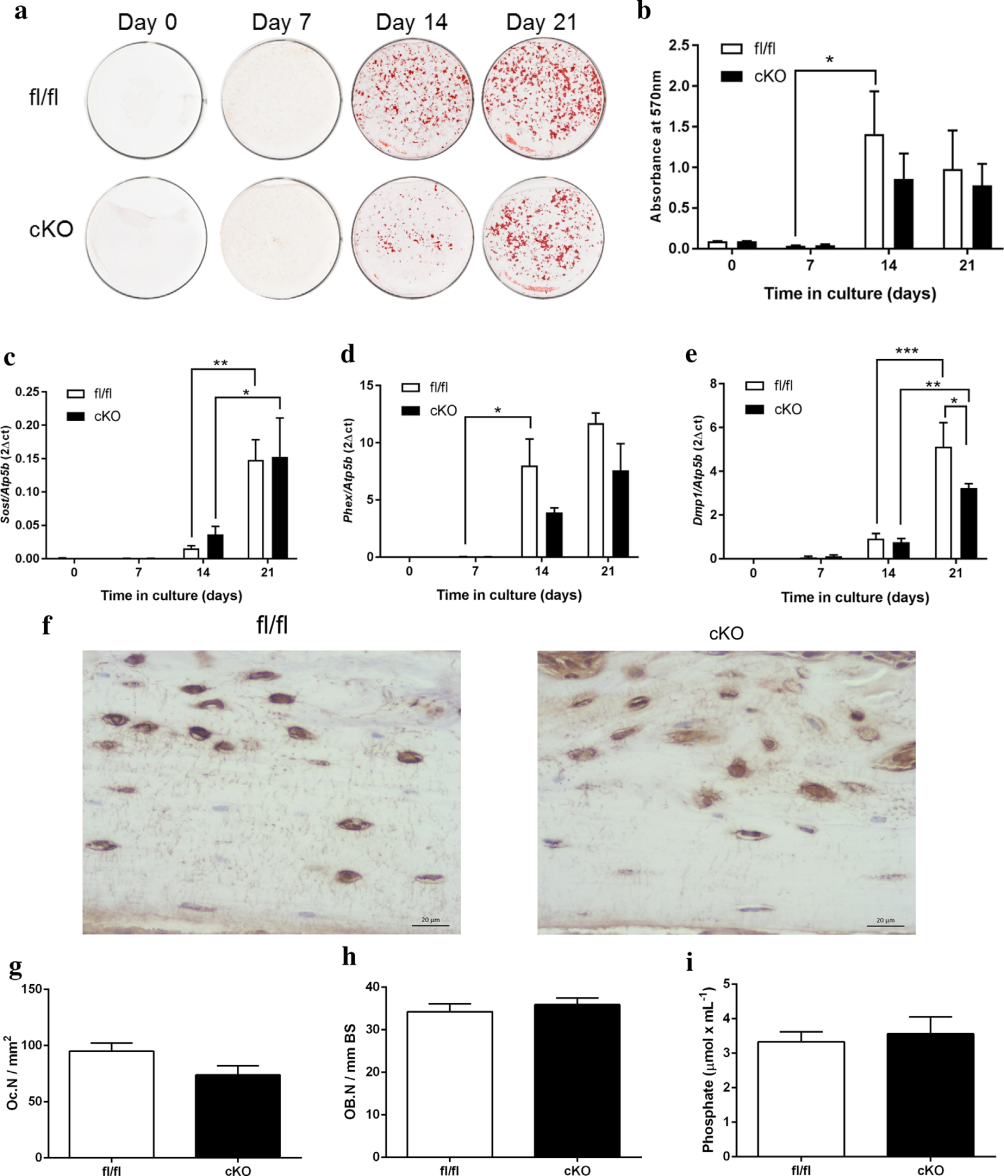
(a) Alizarin red staining of primary osteoblast cultures from Pdpn cKO and fl/fl mice over a 21‐day culture period. (b) Quantification of Alizarin red staining. RT‐qPCR analysis of osteocyte marker genes (c) *Sost*, (d) *Phex*, and (e) *Dmp1* in mineralizing primary osteoblast cultures from Pdpn cKO and fl/fl mice over a 21‐day culture period. (f) Immunohistochemical labeling of sclerostin in the cortical bone of 6‐week‐old mice. Arrows are pointing at embedded osteocytes within the cortical bone and their dendritic processes projecting from the cell bodies. Images are representative of *n* = 4/sex/genotype. Scale bar = 20 μm. (g) Osteoclast cell numbers (OC.N/mm^2^) in the trabecular bone of 6‐week‐old cKO and fl/fl mice (*n* = 4/genotype). (h) Osteoblast cell numbers (OB.N/mm bone surface [BS]) in the trabecular bone of 6‐week‐old cKO and fl/fl mice (*n* = 4/genotype). (i) Serum phosphate levels in cKO and fl/fl mice (*n* = 6/genotype). Data are represented as mean ± SEM. *p < 0.05, **p < 0.01, ***p < 0.001

To examine how Pdpn deletion affects osteocytogenesis, we examined the mineralization and differentiation potential of cultured primary calvaria osteoblasts from Pdpn cKO and fl/fl mice. Alizarin red staining (Figure 4a) and subsequent quantification (Figure 4b) revealed no significant differences in the mineralization capabaility of Pdpn cKO primary osteoblasts in comparison to fl/fl mice. Consistent with this, we observed no significant differences in the gene expression of the osteocyte markers *Sost* and *Phex* in our Pdpn cKO cells at days 0, 7, 14 and 21 of culture (Figure 4c,d). Similarly, no significant differences in *Dmp1* expression were observed at days 0, 7 and 14 although at day 21 of culture, there was a small significant decrease in the expression of *Dmp1* in our Pdpn cKO cultures (*p* < 0.05; Figure 4e). Taken together, these data suggest that the bone selective hypomorphic deletion of Pdpn does not influence osteocyte differentiation.

Due to the proposed role for Pdpn in osteocyte dendrite formation and elongation, we sought to determine whether *Pdpn* deletion modifies osteocyte organization by measuring 3D morphometric parameters in high‐resolution images obtained from nano‐CT at the tibia–fibula junction. Our morphometric evaluation of the cortical bone microarchitecture at the tibia–fibula junction shows that *Pdpn* deletion does not affect numbers of osteocyte lacunae (N.Lc/Ct.TV; Table 3) or lacunar volume (Lc.V/Ct.TV; Table 3). Interestingly, no significant effect on vascular porosity; canal number (N.Ca; Table 3), density (N.Ca/Ct.TV; Table 3) and volume (normalized by cortical tissue volume; Ca.V/Ct.TV; Table 3) was also evident in cKO bones.

**Table 3:** Porosity parameters representing lacuna and vascular porosity of fl/fl and cKO mice detailing the effect of genotype

Morphometric index	fl/fl *n* = 4	cKO *n* = 4	Effect of genotype
Bone parameter			
Ct.TV (mm^−3^)	0.101 ± 0.017	0.078 ± 0.003	NS
Canal parameters			
N.Ca	52 ± 14.634	34 ± 12.124	NS
N.Ca/Ct.TV (mm^−3^)	530 ± 169.45	419 ± 134.24	NS
Ca.V/Ct.TV (%)	0.005 ± 0.001	0.003 ± 0.001	NS
Lacunae parameters			
N.Lc	1792 ± 353	1037 ± 383	NS
N.Lc/Ct.TV (mm^−3^)	18611 ± 4505	13083 ± 4497	NS
Lc.V/Ct.TV (%)	0.004 ± 0.000	0.003 ± 0.000	NS

Data represent means ± SEM with group sizes of *n* = 4 for fl/fl and cKO mice.

**Figure 5:**
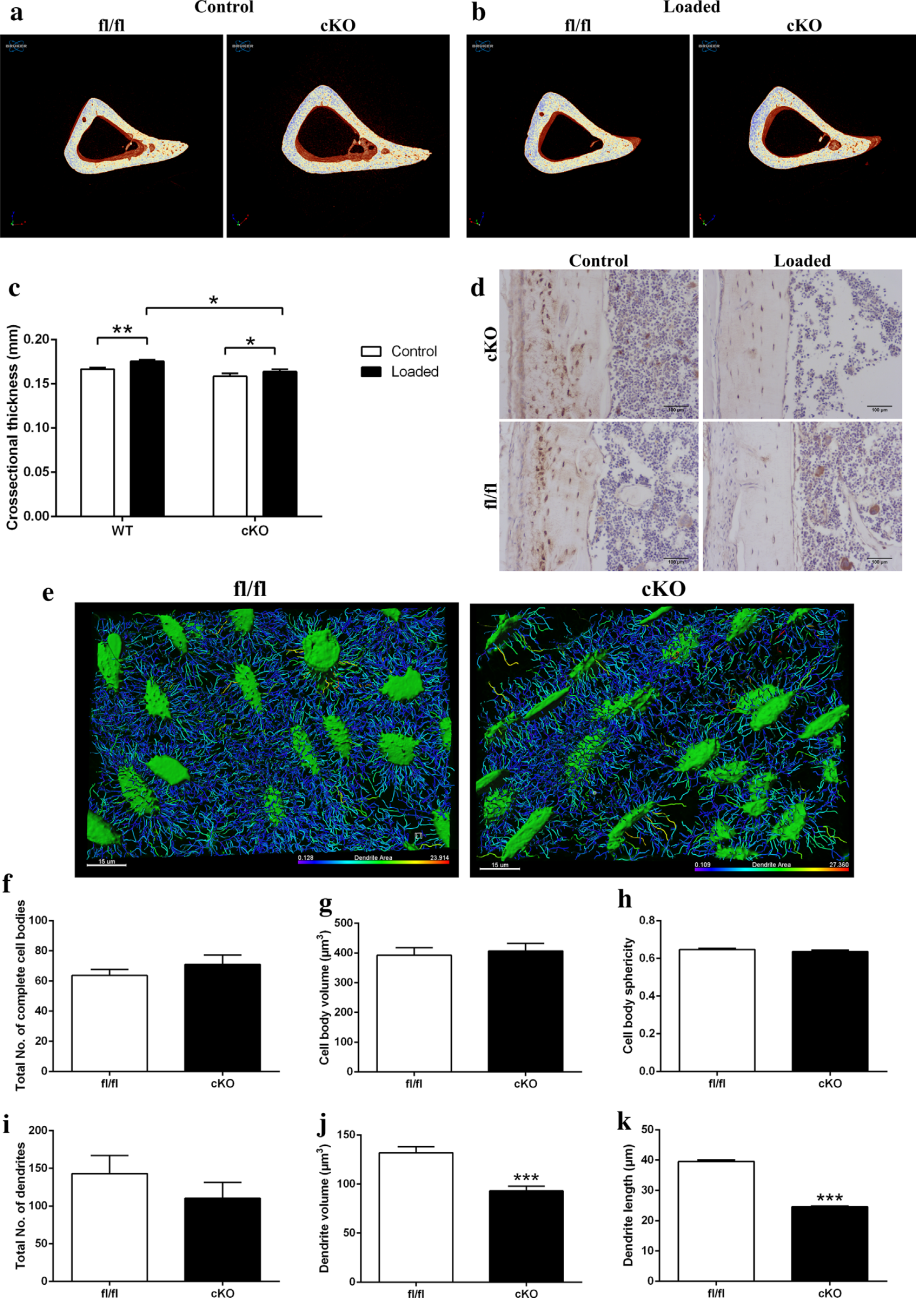
Representative images of fl/fl and cKO mice in (a) control and (b) loaded limbs. (c) Cortical cross‐sectional thickness at 37% of the tibia length as assessed by microCT analysis. (d) Immunohistochemical labeling of sclerostin in osteocytes of cortical bone of loaded and control 6‐week‐old Pdpn cKO and fl/fl mice. Arrows are pointing at embedded osteocytes within the cortical bone and their dendritic processes projecting from the cell bodies. Images are representative of *n* = 4/sex/genotype. Scale bar = 20μm. (e) Laser confocal z‐stack, single channel outlining phalloidin‐Factin staining was imaged in the cortical bone. Imaris cell surface rendering was applied to cell bodies and Imaris FilamentTracer applied to dendritic processes and these were colored according to length. Quantification of osteocyte parameters in 6‐week‐old male fl/fl, and cKO. (f) Total number of complete cell bodies in three volume fields. (g) Cell body volume (μm^3^). (h) Cell body sphericity. (i) Total number of dendrites in three volume fields. (j) Dendrite volume (μm^3^). (k) Dendrite length (μm). Data are represented as mean ± SEM. *p < 0.05, **p < 0.01, ***p < 0.001

Due to the reported function of the osteocyte in regulating bone remodeling and phosphate homeostasis, we next sought to examine whether our cKO mice exhibit differential expression of the osteocyte factor sclerostin, as well as serum phosphate. We observed no apparent differences in the expression of sclerostin in our cKO mice in comparison to fl/fl mice (Figure 4f). Similarly, our assessment of osteoclast and osteoblast numbers by TRAP and H&E staining, respectively and found no significant differences in their number between cKO and fl/fl mice (Figure 4g,h). Serum phosphate levels were also similar in cKO and fl/fl mice (Figure 4i). The osteocyte and its canalicular‐lacunar organization also have another pivotal role in the bone's adaptive response to mechanical stimuli. We therefore next sought to examine whether tibiae deficient in *Pdpn* exhibit a modified response to mechanical loading. As expected, and in line with previous data, fl/fl mice show a significant load‐induced increase in cortical bone cross‐sectional thickness at the proximal diaphysis region of the tibia (*p* < 0.01, Figure 5a–c). Although the scale of the load‐induced increase was statistically smaller than in fl/fl mice (*p* < 0.05), cKO mice also exhibited a significant increase in the tibial cross‐sectional thickness in response to loading (*p* < 0.05, Figure 5a–c; compared to contralateral control). As expected, in both our fl/fl and cKO mice, a decrease in sclerostin immunolabeling was observed in cortical bone osteocytes of the loaded limb in comparison to osteocytes of the unloaded contralateral limb (Figure 5d). There were, however, no differences in osteocyte sclersotin expression between loaded bones from fl/fl and cKO mice (Figure 5d).

In the light of the relatively normal osteocyte organization in cKO mice as disclosed by nano‐CT (Table 3), the difference in response of cKO and fl/fl control mice to mechanical loading was somewhat surprising. Therefore, to determine whether this deficit in load‐related bone adaptation was associated with any specific change in osteocyte morphology, we next performed phalloidin staining of F‐actin in fl/fl and cKO cortical bone. Subsequent 3D rendering (Figure 5e) and quantitative analysis of osteocytes in the diaphyseal cortical bone confirmed our nano‐CT data and revealed no significant differences in the total number of osteocyte cell bodies (Figure 5f), the osteocytes cell body volume (Figure 5g), or the osteocyte cell body sphericity (Figure 5h) in cKO bone in comparison to age‐matched fl/fl bone. Similarly, no significant differences were observed in the number of dendrites formed (Figure 5i). However, a significant decrease in the mean dendrite volume (Figure 5j, *p* < 0.001) and the dendrite lengths (Figure 5k, *p* < 0.001) was noted in cKO in comparison to fl/fl mice, suggestive of a role for Pdpn in attainment of fully developed dendrites.

## 3 DISCUSSION

We, and others, have previously shown that Pdpn promotes osteocytogenesis and dendrite formation in vitro (Staines, Prideaux, et al., 2016; Zhang et al., 2006). Our studies, described herein, have for the first time successfully generated a bone‐specific conditional Pdpn hypomorphic mouse and we have confirmed a role for Pdpn in the attainment of fully elongated osteocyte dendrites.

Previous attempts to decipher the in vivo role of Pdpn in osteocytogenesis have involved global deletion of *Pdpn* in mice. In the lung, Pdpn is known as T1alpha or RT140 and it is expressed on the apical surface of the lung epithelial cells (Dobbs, Williams, & Gonzalez, 1988; Rishi et al., 1995). In mice, global deletion of this gene results in death at birth due to respiratory failure. This is associated with the failure of type II alveolar lung cells to differentiate into type I cells and as such, the lungs of these mice are unable to inflate as normal (Ramirez et al., 2003). T1alpha deletion in mice also produces lymphatic defects with pronounced lymphedema resulting in swelling of the limbs at birth (Schacht et al., 2003). A previous comprehensive study has attempted to analyze the effect of *Pdpn* deletion on the skeleton by generating a global *Pdpn* knock‐out mouse by targeting exon 1 (Zhang et al., 2006). While these mice did not express Pdpn in bone, the animals died soon after birth from suspected lung defect. Although the authors were unable to study the effects of *Pdpn* deletion on the postnatal skeleton, they were able to make some important observations. The general appearance of the embryonic *Pdpn* null and wild‐type mice was similar. More specifically, they found no significant differences in the length or diameter of the femur, or in the femur cortical thickness. They did report an increase in the body weight of the *Pdpn* null mice, however this was attributed to lymphedema resulting from limb swelling (Zhang et al., 2006).

By using the cre‐LoxP system targeted to exon 3 of the *Pdpn* gene, we have generated bone‐specific conditional knockdown mice which exhibited survival indistinguishable from that of fl/fl control mice. This has for the first time allowed us to study the role of Pdpn in bone development in the post‐natal mouse. The normal survival rates of cKO mice contrasts with perinatal lethality of global *Pdpn^‐/‐^* mice (Zhang et al., 2006). Here, we used the well characterized osteocalcin‐driven cre promotor to drive *Pdpn* deletion. Oc‐cre expression has previously been reported to be exclusive to late osteoblasts, with onset of expression just before birth and continuing throughout the mature osteoblast lineage (Zhang et al., 2002). This has therefore allowed us to examine the structure and function of the postnatal skeleton in the presence of reduced Pdpn expression in bone, where we observed significant differences in tibial cortical bone microarchitecture and in the volume and length of osteocyte dendrites likely leading to downstream effects on the tibia's anabolic response to loading.

Osteocytes play a vital role in regulating bone remodeling and their vast dendritic network is critical to cell–cell communication, maintaining cell viability and allowing the transfer of nutrients and waste products (Dallas et al., 2013). Accumulating evidence has suggested that Pdpn may play a critical role in dendrite formation; in MLO‐Y4 cells, the deletion of *Pdpn* with siRNAs abrogated dendrite formation (Zhang et al., 2006). Conversely, the ectopic overexpression of *Pdpn* in keratinocytes induces plasma membrane extensions (Scholl, Gamallo, Vilaro, & Quintanilla, 1999), and in endothelial cells, the reorganization of the actin cytoskeleton and the formation of long tube like structures (Schacht et al., 2003). Similarly, we have previously shown that stabilization of Pdpn protein, through inhibition of endogenous proteasome activity, promotes dendrite formation through the RhoA/ROCK/ERM pathway (Staines, Prideaux, et al., 2016). RhoA is a small GTPase and a master regulator of various cellular processes such as cytokinetics, cytoskeletal regulation, and cell migration (Takai, Sasaki, & Matozaki, 2001). Pdpn is able to co‐localise with the ezrin, radixin and moesin (ERM) family of proteins which are essential for the linkage of the actin cytoskeleton to the plasma membrane (Martín‐Villar et al., 2014, 2006; Scholl et al., 1999). Our present data suggests that Pdpn deletion does not affect osteocyte differentiation and instead, strongly support a role for Pdpn in the formation of full length dendritic processes. Despite this, we did not observe any differences in the expression of factors involved in the osteocytic regulation of bone remodeling and phosphate homeostasis. Normal sclerostin expression in osteocytes within bone of Pdpn cKO mice is consistent with a lack of change in osteocyte number, shape and size in these mice. It is possible that the hypomorphic deletion of Pdpn expression in osteocytes does not affect the osteocyte's ability to regulate bone remodeling. Rather, the decreased magnitude of bone accrual in response to mechanical loading is likely a consequence of inadequate dendrite formation and a lack of critical osteocyte mechanoregulatory function.

Mechanosensation and mechanotransduction were the earliest functions ascribed to the osteocyte and since then, there has been a wealth of evidence defining their role and in particular, the role of the dendritic network in sensing mechanical loads. In recent years, the complex biochemical pathways through which the osteocyte converts its mechanical strain signals into a biological output have been unravelled. These include intracellular calcium, nitric oxide, ATP, and prostaglandins and these have direct effects on osteoblasts to alter the bone microarchitecture (Dallas et al., 2013). On this basis, we therefore speculate that the restricted osteocytic dendrite network observed in our hypomorphic Pdpn cKO mice would likely lead to altered bone geometry and microarchitecture; the reduced dendritic network impairs the ability of the osteocyte to sense load and convert this to a biological signal affecting osteoblast function. It would obviously therefore be of interest to examine the expression of the aforementioned biochemical pathways in our Pdpn cKO mice in response to loading. Moreover, as the bone mechanical properties, including stiffness, depend upon the geometry of the bone shaft, it is likely that the reduction of the osteocyte dendritic network affects the mechanical properties as a secondary effect through these changes in the microarchitecture (Saffar, Jamilpour, & Rajaai, 2009).

Here, we used whole bone μCT analysis to fully define these changes in microarchitecture and determine the estimated strength and rigidity of the bone through the study of the bone's cross sectional geometry. CSA is directly related to a bone's strength against compressive forces applied equally throughout the bone; factors such as bone shape and the effects of muscle contraction, however, result in long bones experiencing bending and torsional forces (Javaheri et al., 2015). We measured indices of rigidity, including maximum and minimum resistance against bending forces in the cross section, second moment of area around minor axis (I_min_), and second moment of area around major axis (I_max_) in male and female cKO tibia and their age‐matched control mouse tibia. Our data, using this approach, revealed sex‐dependent differences in cortical bone microarchitecture and in our estimation of tibial resistance to bending forces in cKO mice in comparison to control mice. These differences suggest that bone responses to dysfunctional osteocyte form and function may be sex‐dependent. Similarly, the differences in the regional responses in both male and female bones suggest that *Pdpn* may play differential roles in bone development and function. Interestingly, Bonewald and colleagues found osteocyte Pdpn expression was increased in an ulna loading model, but that this increase was not consistent along the length of the diaphysis (Zhang et al., 2006). This regional requirement for *Pdpn* function during physiological loading in the post‐natal mouse may explain the differences noted in structural parameters of the *Pdpn* cKO mouse and in the relatively minor, albeit significant, reductions in load‐related increases in cortical thickness in the bones of these mice.

Despite these changes in bone microarchitecture and osteocyte form in our *Pdpn* cKO mice, our data reveal no effect of *Pdpn* deletion on the gross phenotype. This suggests that while *Pdpn* plays a role in dendrite length and bone microarchitecture, there may be redundancy associated with the function of *Pdpn*. Possible candidates include Dentin matrix protein 1 (DMP1). DMP1 is an acidic phosphorylated extracellular matrix protein that is part of the Small; Integrin‐Binding LIgand, N‐linked Glycoprotein (SIBLING) family along with bone sialoprotein (BSP), osteopontin (OPN), dentin sialophosphoprotein (DSPP), and matrix extracellular phosphoglycoprotein (MEPE). All of these family members contain an RGD sequence for integrin binding and can bind to hydroxyapatite (Staines, Macrae, & Farquharson, 2012). DMP1 is highly expressed by osteocytes and is restricted to the dendritic processes. Deletion of DMP1 in mice causes remarkable defects in both tooth and bone (Ye et al., 2004, 2005). DMP1 knockout mice also display an abnormal lacuna‐canalicular system, with a reduction in the number of canaliculi and a twofold expanded osteocyte lacunae with rough, not smooth, lacunar walls (Lu et al., 2011). The authors of this elegant study also showed that this phenotype can be rescued by the re‐expression of the 57‐kDa C‐terminal fragment of DMP1 (Lu et al., 2011). DMP1 has been demonstrated to bind to the hyaluronan receptor CD44, a membrane bound protein thought to interact with the ERM family of proteins that are involved in actin cytoskeleton rearrangement and as such, the formation of the osteocyte dendritic processes (Jain, Karadag, Fohr, Fisher, & Fedarko, 2002).

As we did not achieve complete cre‐recombination and consequently not a complete knock‐out of *Pdpn* expression (∼70% reduction), it is also possible that a low level of *Pdpn* expression is sufficient to drive osteocytogenesis. A more complete knock‐out of *Pdpn* expression may be required to reduce dendrite formation to a level where bone function is pathologically compromised. Our decision to use the osteocalcin‐cre promotor mouse to drive deletion of Pdpn selectively in bone was based on the onset of OCN expression. Despite this, there have been previous reports of incomplete recombination when using these mice (Xiao et al., 2010). As such it might be prudent to attempt another cre driver mouse. Such examples would include the col 2.3 kb (Col 2.3‐Cre) and 3.6 kb (Col 3.6‐Cre) fragments of the rat Col1a1 promoter or the osterix‐cre mouse (Liu et al., 2004; Rodda & McMahon, 2006).

In summary, our data confirm a role for *Pdpn* in the formation of full length dendritic processes during osteocytogenesis and suggest that dysfunctional osteocyte dendrite formation is sufficient to alter the bone microarchitecture. The generation of a viable post‐natal bone specific *Pdpn* cKO will enable future research into understanding the importance of *Pdpn* in osteocyte function, and in particular to the in vivo effects of dysfunctional osteocyte formation on bone homeostasis.

## 4 MATERIALS AND METHODS

### 4.1 Generation of a Pdpn conditional knockout mouse

We obtained floxed *Pdpn* mice from the EUCOMM/KOMP, MRC Harwell, Oxfordshire, UK and OC‐cre mice as a kind gift from Thomas Clemens at John Hopkins Medicine, Baltimore, Maryland (Zhang et al., 2002). *Pdpn* floxed mice were designed with the *loxP* sites around exon 3 (Figure 1a). Mice were crossed to generate Oc‐Cre;*Pdpn* ^flox/flox^ conditional knockout mice (cKO) as well as appropriate *Pdpn* ^flox/flox^ control mice (fl/fl). PCR‐based genotyping was performed on mouse DNA using a duplex PCR reaction for *Cre* (F: GCA TTA CCG GTC GAT GCA ACG AGT GAT GAG; R: GAG TGA ACG AAC CTG GTC GAA ATC AGT GCG) and *Fabpi200* (F: TGG ACA GGA CTG GAC CTC TGC TTT CCT AGA; R: TAG AGC TTT GCC ACA TCA CAG GTC ATT CAG) and for *Pdpn* (F: TCC CAC ACC AGG TTT TGT GT; R: CAG TGA GCC ATC TCT CCA GC). Mice were kept in polypropylene cages, with light/dark 12‐hr cycles, at 21 ± 2°C, and fed ad libitum with maintenance diet (Special Diet Services, Witham, UK). All experimental protocols were approved by Roslin Institute's Animal Users Committee and the animals were maintained in accordance with UK Home Office guidelines for the care and use of laboratory animals. All analysis and data acquisition were completed on 6‐week‐old mice and tissue obtained from them.

### 4.2 Determination of Cre‐mediated recombination efficiency

To determine the specificity and efficiency of Cre‐mediated recombination, PCR analysis was performed on genomic DNA extracted from bones using a Qiagen, Manchester, UK DNA extraction kit according to the manufacturer's instructions. Primers were specific for alleles before recombination (Tm1c; F: TGG AAT GGC TGT GAG TTC TG; R: CTA AAA TGG AGT TGG AGA TGG ATA C) and after recombination (Tm1d; F: TGA GCG AGC AGA GGT CCT AA; R: GCG TCT GGC ACT CTC AGA AG).

### 4.3 Primary osteoblast isolation and culture

Primary calvarial osteoblasts were obtained from 3‐day‐old Pdpn cKO and fl/fl mice by sequential enzyme digestion of excised calvarial bones using a four‐step process as has previously been described (Staines, Zhu, Farquharson, & Macrae, 2013) (1 mg/ml collagenase type II in Hanks' balanced salt solution [HBSS] for 10 min; 1 mg/ml collagenase type II in HBSS for 30 min; 4 mM EDTA for 10 min; 1 mg/ml collagenase type II in HBSS for 30 min). The first digest was discarded and the cells were resuspended in growth medium consisting of a‐MEM (Invitrogen, Paisley, UK) supplemented with 10% (v/v) FCS (Invitrogen) and 1% gentamycin (Invitrogen). Osteoblasts were seeded at a density of 1 × 10^4^ cells/cm^2^ and cultured for up to 21 days with the addition of 2.5 mM β‐glycerophosphate and 50 µg/ml ascorbic acid. At days 0, 7, 14, and 21, cells were either processed for RNA extraction or fixed in 4% paraformaldehyde and stained with 2% alizarin red (pH 4.2) for 5 min at room temperature. Alizarin red‐stained cultures were extracted with 10% cetylpyridinium chloride for 10 min and optical density was measured at 570 nm.

### 4.4 Gait analysis

Gait parameters of freely moving male mice were measured using the CatWalk gait analysis system (Noldus Information Technology, the Netherlands) as described previously (Hamers, Lankhorst, van Laar, Veldhuis, & Gispen, 2001; Masocha & Parvathy, 2009). Each mouse was placed individually in the CatWalk walkway and allowed to walk freely and traverse from one side to the other of the walkway glass plate. Mice were habituated every day for 2 weeks prior to the test run, in which the gait of all mice was recorded three times and analyzed using the CatWalk system. Analysis of the recording generated a wide range of parameters; those analyzed are detailed in Supplementary Table S1.

### 4.5 In vivo loading

Twelve‐week‐old male mice were isoflurane‐anesthetized and the right tibia loaded as described previously using a well‐established model for comparing architectural load‐induced changes in tibiae in control and mutant mice in which the contralateral left tibia is used as control (De Souza et al., 2005). Briefly, axial compressive loads were applied by a servo‐hydraulic materials testing machine (Bose, Framingham, Massachusetts, UK) via custom‐made cups which hold knee and ankle joints flexed and the tibia vertically. The loading pattern consisted of a trapezoidal wave, with peak 11N loads for the cKO and 12N for the WT mice for 0.05 s, rise and fall times 0.025 s each and baseline hold time of 9.9 s at 2 N (calibrated for peak strain level by finite element analysis) (Pereira, Javaheri, Pitsillides, & Shefelbine, 2015). Forty cycles were applied in each loading episode. Twelve‐week‐old male cKO and WT mice (*n* > 3/genotype) were loaded three times per week for 2 weeks and left and right tibia dissected 3 days after the final loading episode.

### 4.6 Whole mount staining

Male mice were euthanized by CO_2_ and incubated for 24 hr in tap water. Carcasses were then scalded in hot tap water to enable removal of the skin, skinned, and eviscerated, and then fixed in 95% ethanol for 3–5 days. The preparations were then incubated in 0.015% Alcian blue (pH 0.75) for 24 hr to stain sulphated glycosaminoglycans in the cartilage, rinsed twice in 95% ethanol, and fixed in 95% ethanol for another 2 days. Mice were then cleared of the remaining muscle by a 6‐hr incubation in 1% KOH (w/v) and the bone was stained with fresh 0.005% Alizarin Red for 3 hr. Mice were then further cleared in 2% KOH (w/v) for 48 hr followed by decreasing concentrations of 2% KOH (w/v) in glycerol for 24 hr each (80:20, 60:40, 40:60, and 20:80, respectively). The skeletal preparations were stored in 20:80 2% KOH:glycerol at room temperature.

### 4.7 Tissue processing for histological techniques

Soft tissues (liver, spleen, lung, heart, and kidney) and tibia/femurs were dissected from freshly killed male and female mice and fixed in 4% paraformaldehyde (PFA) for 24 hr at 4°C. Tibiae were subsequently decalcified in 10% ethylenediaminetetracetic acid (EDTA/PBS, pH 7.4 at 4°C; Sigma–Aldrich, Dorset, UK) for 3–4 weeks with regular changes. Soft tissues and legs were dehydrated and processed to paraffin wax using standard procedures. Sections (5 μm) were cut and used for histological and immunohistochemical analysis. To analyze osteoclast and osteoblast numbers, sections were reacted for tartrate‐resistant acid phosphatase activity (TRAP) and stained by H&E respectively, as described previously (Erlebacher & Derynck, 1996). Osteoclasts were quantified per mm^2^ of trabecular bone, and osteoblasts per mm of bone surface, in an identical region at an equivalent distance beneath the growth plate in all samples.

### 4.8 Immunohistochemistry

For immunohistochemical analysis, sections were dewaxed in xylene and rehydrated. Sections were incubated at 37°C for 30 min in 1 mg/ml trypsin for antigen demasking. Endogenous peroxidases were blocked by treatment with 3% H_2_O_2_ in methanol (Sigma). Ppdn antibodies (Polyclonal raised in Goat IgG; R&D systems, Abingdon, UK) were used at a dilution of 1/100; and sclerostin antibodies (Polyclonal raised in Goat IgG; R&D systems) at a dilution of 1:200 with appropriate IgG controls. The Vectastain ABC universal kit (Vector Laboratories, Peterborough, UK) was used according to the manufacturer's instructions. The sections were dehydrated, counterstained with hematoxylin, and mounted in DePeX.

### 4.9 Phalloidin staining

Femurs from male mice were decalcified as described above and then cryoprotected in 30% sucrose (w/v) at 4°C for 48 hr. The femora were cut in the mediolateral plane in serial longitudinal 20‐μm thick sections using a cryostat and thaw‐mounted on gelatin‐coated slides for processing. Slides were dried at room temperature for 45 min, washed in PBS twice for 5 min each, and incubated with 0.1% Triton‐X 100 (Sigma–Aldrich) for 30 min and then rinsed with PBS. Slides were then incubated with Alexa Fluor 488‐conjugated phalloidin (1:20; Life Technologies, Grand Island, NY) for 1 hr. Bone sections were washed in PBS and mounted in VectaShield (Vector Laboratories). Preparations were allowed to dry at room temperature for 12 hr.

### 4.10 Analysis of phalloidin staining

Sections were imaged on a Zeiss LSM 710 Laser Scanning Confocal Microscope with 488 nm laser excitation and detection settings from 493 nm to 634 nm. Z‐stacks were produced with optimal Nyquist overlap settings using a 63×/1.4na oil immersion lens. Voxel sizes were 0.1 × 0.1 × 1.00 μm in *x*,*y*,*z* planes. respectively. A comparable region of interest was analyzed for osteocyte parameters in all samples located in the diaphyseal cortical bone. Image stacks were imported into Bitplane Imaris 8.2.0 software and algorithms were created with Imaris FilamentTracer to render and measure dendritic processes. Surface rendering was used for osteocyte cell body measurements.

### 4.11 Micro‐computed tomography (μCT) scanning

Tibiae from male and female mice were dissected and frozen at −20°C until required. Scans were performed with an 1172 X‐Ray Microtomograph (Skyscan, Belgium) to evaluate bone geometry. High‐resolution scans with an isotropic voxel size of 5 μm were acquired (50 kV, 200 μA, 0.5 mm aluminum filter, 0.6° rotation angle, two frame averaging, exposure time 1650). The projection images were reconstructed using NRecon software version 1.6.9.4 (Skyscan, Belgium) to produce tomograms that underwent processing during reconstruction to correct for beam‐hardening and ring artifacts.

### 4.12 Morphometrical analysis

#### 4.12.1 Trabecular bone

For the trabecular analysis, the base of the growth plate was used as a standard reference point. Trabecular bone was analyzed in a 1‐mm region of the proximal tibia 250 μm from this reference point (toward the diaphysis). Data were analyzed with CtAn software (Skyscan) as previously described (Staines, Madi, et al., 2016).

#### 4.12.2 Whole bone cortical μCT analysis

Whole bone analysis was performed on datasets derived from whole CT scans using BoneJ (version 1.13.14) a plugin for ImageJ, as previously described (Javaheri et al., 2015). Following segmentation, alignment, and removal of fibula from the dataset, a minimum bone threshold was selected using a histogram‐based method in ImageJ which utilizes all pixels in a stack to construct a histogram and was further confirmed using ImageJ “threshold function.” The gray level threshold ranged between 22,000 and 22,100 and was applied to all datasets to separate higher density bone from soft tissues and air. This threshold was used in “Slice Geometry” function within BoneJ to calculate bone cross sectional area (CSA), second moment of area around the minor axis (I_min_), second moment of area around the major axis (I_max_), mean cortical thickness determined by local thickness in two dimensions (Ct.Th), ellipticity and resistance to torsion (J). The most proximal and distal (10%) portions of the tibial length were excluded from analysis, as these regions include trabecular bone.

### 4.13 Nano‐computed tomography analysis

The samples were placed in Orthodontic Wax (Kerr, CA) at 50 kV and 200 µA, 9,800‐ms exposure time with a 0.25‐mm aluminum filter (99.999% purity, Goodfellow, Huntington, UK), voxel size of 0.6 µm, 360° at a rotation step of 0.25°. Two‐frame averaging was used to improve the signal‐to‐noise ratio. We analyzed 300 consecutive images from the tibia–fibula junction from each sample. Using the CtAn software, osteocyte lacunar indices were calculated by measuring the 3D parameters of each discreet object within the volume of interest after segmentation as described previously (Javaheri et al., 2015). Shape analysis of the lacunae was conducted utilizing “Analyze Particles” function in BoneJ.

### 4.14 Mechanical testing

A Lloyd LRX5 materials testing machine (Lloyd Instruments, West Sussex, UK) fitted with a 100 N load cell was used to determine bone stiffness and point of failure of tibiae. The span was fixed at 10  mm, and the cross‐head was lowered at 1  mm/min. Data were recorded after every 0.2‐ mm change in deflection. Each bone was tested to failure, with failure points being identified as the point of maximum load from the load–extension curve. The maximum stiffness was defined as the maximum gradient of the rising portion of this curve (Huesa et al., 2011).

### 4.15 RNA extraction and quantitative real‐time PCR (RT‐qPCR)

Total RNA was extracted using the RNEasy mini kit (Qiagen) according to the manufacturer's instructions. RNA samples were reverse‐transcribed into cDNA using Superscipt II reverse transcriptase (Invitrogen). RT‐qPCR was carried out in a Stratagene Mx3000P cycler with each reaction containing 50‐ng template DNA, 250 nM forward and reverse primers (Primer Design, Southampton UK) (*Sost*: F: TGAGAACAACCAGACCATGAAC, R: TCAGGAAGCGGGTGTAGTG; *Dmp1*: F: ATACCACAATACTGAATCTGAAAGC, R: CACTATTTGCCTGTCCCTCTG; *Phex*: F: CTAACCACCCACTCCCACTT, R: CCAATAGACTCCAAACCTGAAGA; Atp5b: sequences not available) and PrecisionPlus Mastermix (Primer Design). The Ct values for the samples were normalized to that of GAPDH and the relative expression was calculated using the ΔΔCt method (Livak & Schmittgen, 2001). The amplification efficiencies of all the primers were between 90% and 100%.

### 4.16 Western blotting

Femurs from male mice had their epiphyses removed and were flushed to remove bone marrow, snap frozen, and lysed in RIPA buffer (Sigma), containing protease inhibitors (Roche, Burgess Hill, UK). Protein content was determined using the DC protein assay (Bio‐Rad Laboratories, Watford, UK). The lysates were run on 10% Bis–Tris gels. Following transfer, nitrocellulose films were blocked in 5% milk, and probed overnight at 4°C with Pdpn primary antibody (R&D Systems; 1:1,000). The nitrocellulose was subsequently incubated with peroxidase‐labeled rabbit anti‐goat antibodies (Dako, Ely, UK) for a further 90  min. ECL detection reagents were used to visualize bands on hyperfilm (GE Healthcare, Bucks, UK). Where necessary, the nitrocellulose was stripped using Restore Plus Stripping Buffer (GE Healthcare). Densitometry of Western blotting was measured on three independent samples using ImageJ.

### 4.17 Serum phosphate analysis

Immediately before termination, blood was collected and incubated on ice for 30 min, before centrifugation at 15,000 kg for 10 min. The serum supernatant was stored at −80°C until analyzed for phosphate levels using a colorimetric Phosphate Assay Kit (Abcam, Cambridge, UK; 1:100) according to the manufacturer's instructions.

### 4.18 Statistical analysis

Data are expressed as the mean ± SEM of at least three replicates per experiment. Results were analyzed blinded. For cortical bone, graphs were developed using the R programming language “R,” version 3.1.3 (R Foundation for Statistical Computing, Vienna, Austria; http://www.r-project.org). Normality and homogeneity of variance of all the data were checked using the Shapiro–Wilk and the Bartlett's test in R 3.1.3, respectively. Two‐sample Student's *t*‐test was used to compare means between female cKO and fl/fl, and between male cKO and fl/fl. Kruskal–Wallis test was employed if either the normality or the homogeneity of variance assumptions were violated (*p* ≥ 0.05). *p* < 0.05 was considered to be significant and noted as *; *p*‐values of <0.01 and <0.001 were noted as ** and ***, respectively.

## Supporting information

Additional Supporting Information may be found online in the supporting information tab for this article.

**Figure S1**. Gait analysis of 6‐week old male fl/fl and cKO mice (n = 4/genotype/sex). Data are represented as mean ± S.E.M.
**Table S1**. Gait parameters explored in this study.
