# Supplementary material for: Hypomorphic conditional deletion of E11/Podoplanin reveals a role in osteocyte dendrite elongation
Source: J Cell Physiol. 2017 Jun 14;232(11):3006–19. doi: 10.1002/jcp.25999 (PMC5575468; doi:10.1002/jcp.25999)
Supplement: Supplementary file 1 — Figure S1. Gait analysis of 6‐week old male fl/fl and cKO mice (n = 4/genotype/sex). Data are represented as mean ± S.E.M. Table S1. Gait parameters explored in this study. [file JCP-232-3006-s001.pdf]

Hypomorphic conditional deletion of E11/Podoplanin reveals a pivotal role in osteocyte dendrite elongation

Dr. Katherine A. Staines PhD[1,2*]., Dr. Behzad Javaheri PhD[3]., Dr. Peter Hohenstein PhD[1]., Mr. Robert Fleming BSc[1]., Mr Mark Hopkinson BSc[3]., Dr. David J. Buttle PhD[4]., Prof. Andrew A. Pitsillides PhD[3]., Prof. Colin Farquharson PhD[1]

[1] Roslin Institute and R(D)SVS, The University of Edinburgh, Easter Bush, Midlothian, UK EH25 9RG [2] School of Applied Sciences, Edinburgh Napier University, Sighthill Campus, Edinburgh UK EH11 4BN [3] Comparative Biomedical Sciences, Royal Veterinary College, Royal College Street, London, UK NW1 0TU [4] Dept of Infection, Immunity and Cardiovascular Disease, University of Sheffield

* Corresponding author

Katherine A. Staines

School of Applied Sciences

Edinburgh Napier University

Sighitill Campus

Edinburgh EH11 4BN

Email: k.staines@napier.ac.uk

Tel: 0131 455 3369

Suppl Fig 1. Gait analysis of 6-week old male fl/fl and cKO mice (n=4/genotype/sex). Data are represented as mean ± S.E.M

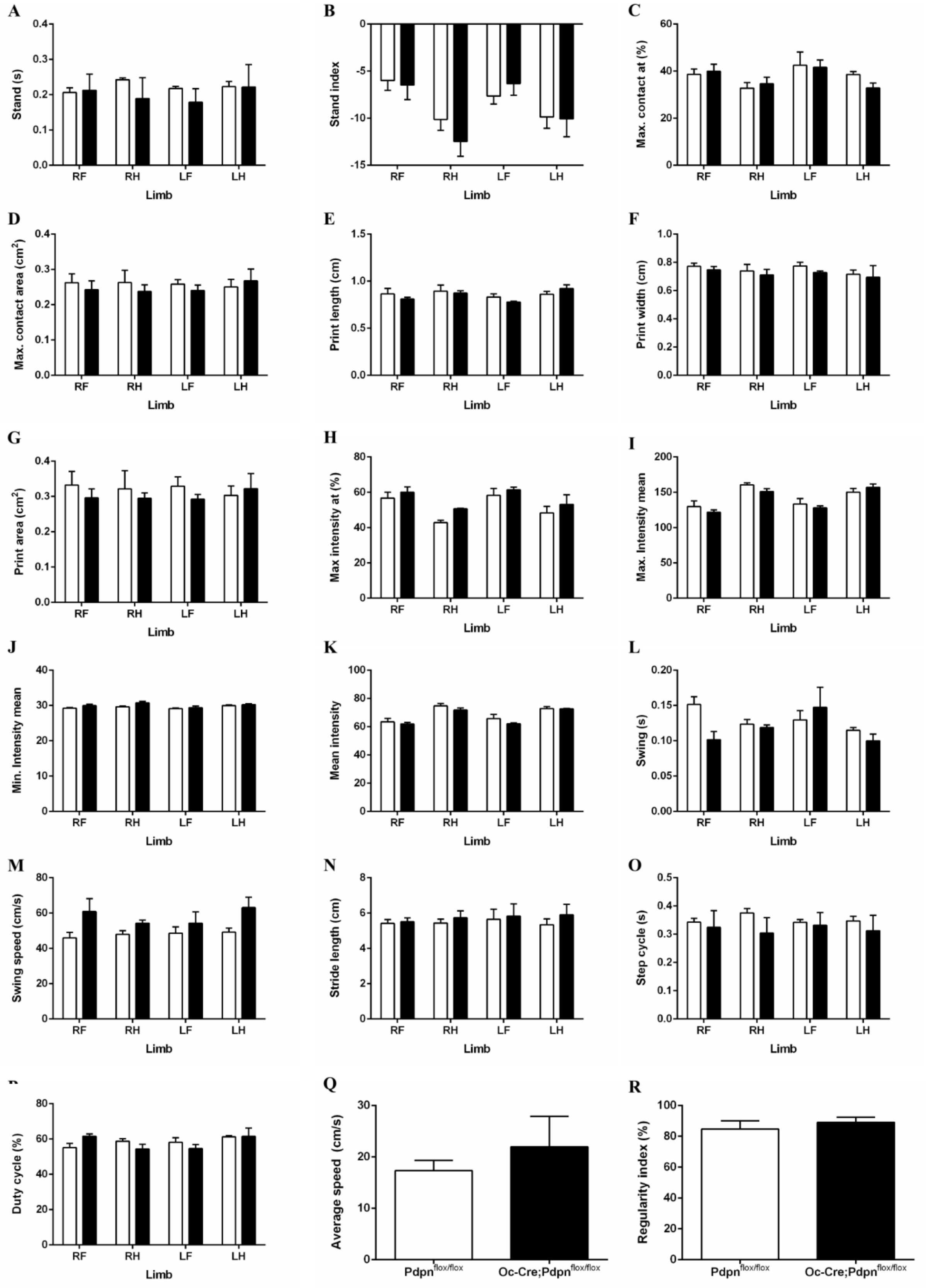

Suppl. Table 1. Gait parameters explored in this study

**Paw Parameter**	**Explanation**
Stand (s)	The duration in seconds of contact of a paw with the glass plate
Stand Index	The speed at which the paw loses contact with the glass plate
Max Contact At (%)	(Max Contact At (s) - Initial Contact At (s))/Stand (s) x100%<br>Max Contact At (s) : the time in seconds since the start of the run that a paw makes maximum contact with the glass plate
Max Contact Area (mm^2)	The maximum area of a paw (in the Distance Unit) that comes into contact with the glass plate
Print Length (mm)	The length (horizontal direction) of the complete print
Print Width (mm)	The width (vertical direction) of the complete paw print
Print Area (mm^2)	The surface area of the complete print
Max Intensity At (%)	(Max Intensity At (s) - Initial Contact At (s))/Stand (s) x100%<br>Max Intensity At (s): the time in seconds since the start of the run that the maximum intensity is measured
Maximum Intensity mean	The mean maximum intensity of the complete paw
Minimum Intensity mean	The mean minimum intensity of the complete paw
Mean Intensity	The mean intensity of the complete paw
Swing (s)	The duration in seconds of no contact of a paw with the glass plate
Swing Speed (cm/s)	The speed of the paw during Swing: Stride Length/Swing
Stride Length (cm)	The distance between successive placements of the same paw
Step Cycle (s)	The time in seconds between two consecutive Initial Contacts of the same paw: Stand + Swing
Duty Cycle (%)	Stand as a percentage of Step Cycle: Stand/(Stand+Swing)x100%
Average speed (cm/s)	The average speed of the run
Regularity index (%)	The number of normal step sequence patterns relative to the total number of paw placements
